# Multiple Regression Methods Show Great Potential for Rare Variant Association Tests

**DOI:** 10.1371/journal.pone.0041694

**Published:** 2012-08-08

**Authors:** ChangJiang Xu, Martin Ladouceur, Zari Dastani, J. Brent Richards, Antonio Ciampi, Celia M. T. Greenwood

**Affiliations:** 1 Lady Davis Institute for Medical Research, Jewish General Hospital, Montreal, Quebec, Canada; 2 Department of Epidemiology, Biostatistics and Occupational Health, McGill University, Montreal, Quebec, Canada; 3 Department of Human Genetics, McGill University, Montreal, Quebec, Canada; 4 Department of Medicine, Jewish General Hospital, McGill University, Montreal, Quebec, Canada; 5 Twin Research and Genetic Epidemiology, Kings College London, London, United Kingdom; 6 Department of Oncology, McGill University, Montreal, Quebec, Canada; University of California, Irvine, United States of Ameirca

## Abstract

The investigation of associations between rare genetic variants and diseases or phenotypes has two goals. Firstly, the identification of which genes or genomic regions are associated, and secondly, discrimination of associated variants from background noise within each region. Over the last few years, many new methods have been developed which associate genomic regions with phenotypes. However, classical methods for high-dimensional data have received little attention. Here we investigate whether several classical statistical methods for high-dimensional data: ridge regression (RR), principal components regression (PCR), partial least squares regression (PLS), a sparse version of PLS (SPLS), and the LASSO are able to detect associations with rare genetic variants. These approaches have been extensively used in statistics to identify the true associations in data sets containing many predictor variables. Using genetic variants identified in three genes that were Sanger sequenced in 1998 individuals, we simulated continuous phenotypes under several different models, and we show that these feature selection and feature extraction methods can substantially outperform several popular methods for rare variant analysis. Furthermore, these approaches can identify which variants are contributing most to the model fit, and therefore both goals of rare variant analysis can be achieved simultaneously with the use of regression regularization methods. These methods are briefly illustrated with an analysis of adiponectin levels and variants in the ADIPOQ gene.

## Introduction

New methods for the analysis of rare genetic variants are appearing rapidly. Resequencing efforts are identifying numerous new variants but the majority of the new variants are seen only in a very small number of individuals [1]. Hence, the new methods for rare variants, in general, look for association between phenotypes and the collection of all rare variants in a defined set, such as all variants in or near a gene [2].

Hoffman [3], and Lin and Tang [4] framed the goal of rare variant statistical analysis as a problem of distinguishing which (if any) of a set of genetic variants are associated with the phenotype. Let 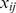 be a genotype coding for the 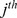 variant in individual 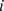, where 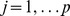, and 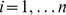. For example, 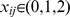 for additive allele coding. Suppose that a phenotype 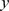 is related to a set of genetic variants by
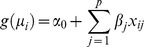
(1)for an appropriate function 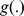, where 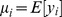 is the mean of 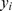. The parameters 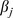 reflect the effect of variant 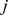 on the phenotype. In this framework, therefore, rare variant analysis is used to answer two questions: (1) Are any of the parameters 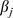 nonzero? (2) If some parameters are nonzero, which ones?

Usually there are very few individuals carrying the minor allele at the majority of the identified variants, and therefore it is extremely challenging to estimate the parameters 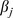 using single marker tests. Joint analysis of a set of genetic variants has therefore been proposed as an alternative strategy to get around this issue of very sparse data. The many proposed methods encompass a wide variety of approaches [5]–[7]. Some approaches assume a “burden” hypothesis where the count of rare variants is associated with increased risk [4], [8], [9]. Others methods assume an increased variance of the phenotype or in the risk distribution in the presence of one or more causal rare variants [9]–[11]. A third group examines genotypic or haplotypic similarities between individuals [9], [12].

Conceptually, the problem of how best to model the relationship between a phenotype and a large set of rare genetic variants is a problem of variable selection (or feature selection) and/or dimension reduction (or feature extraction) in a sparse covariate space. There are many well-studied statistical methods for feature selection and extraction when the number of predictor variables is large. However, for the analysis of rare genetic variation, such approaches have only recently been explored. There were several groups at the GAW17 workshop in 2010 who implemented feature extraction or penalization methods, using a wide variety of different approaches [13]–[18], and a few other publications have appeared recently using such methods (e.g. [19]–[21]). Some groups first collapsed the rare variants, and then implemented a LASSO or PLS model using the common variants and the collapsed rare variants [14], [16], [21]. Others addressed the question of simultaneous modelling across multiple regions or genes, combining methods such as LASSO or PLS first at the gene level, and then across genes [13], [15]. A few publications described innovative approaches specifically developed for the sequencing context: Ayers et al. [17] built a LASSO with three custom penalties encouraging different aspects of shrinkage; Luo et al. [20] combined LASSO with local linear embedding. Each of these papers featured a different multiple regression method.

**Figure 1:**
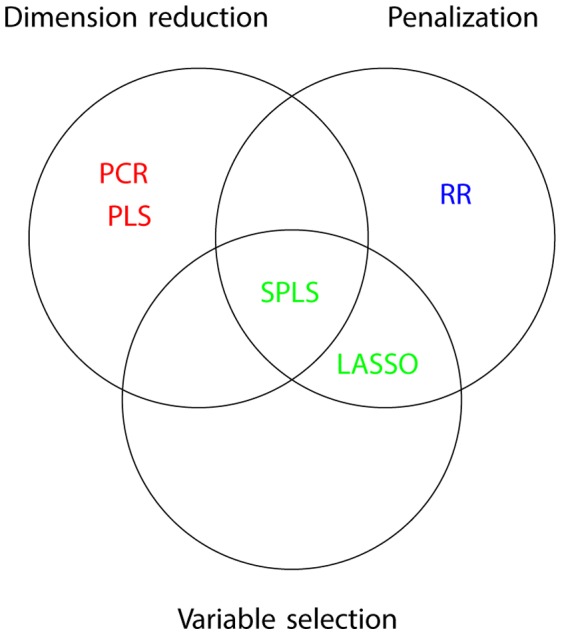
Characteristics of the regression regularization methods compared. The methods are characterized by whether there is variable selection, penalization of parameter estimates, or dimension reduction.

In this paper, we explore whether several classic approaches for feature selection or extraction (ridge regression (RR) [22], LASSO [23], principal components regression (PCR) [24], partial least squares (PLS) regression [25], [26], or sparse PLS (SPLS) [27]) can effectively identify associations between a genetic region and a continuous trait. Features of the five chosen methods are shown in Figure 1 and Table 1, respectively. All penalized regression methods minimize a penalized log-likelihood, so that the regression coefficients are shrunk toward zero. However, methods differ in which penalty functions are used. RR uses the 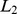-norm penalty, which minimizes the sum of squares of deviations, while LASSO uses 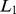-norm penalty, minimizing the absolute value of the deviations. Since the 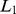 LASSO shrinks some of the coefficients to be exactly zero, it can be considered as a variable selection method (Figure 1). PCR and PLS reduce the dimension of the variable space by constructing linear combinations of the original variables, but the methods differ in how the linear combinations are constructed. In PCR, the transformed variables are chosen to explain as much variance as possible in the predictor variable space. In contrast, PLS features are chosen to have high correlation with the response variable. In both PCR and PLS, the number of transformed variables or features included in the regression model must be chosen. Therefore, these methods can be considered as feature selection methods; the feature selection occurs in the transformed variable space. A sparse PLS model was proposed by adding an 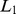 penalty to PLS regression [27]. In SPLS, there is variable selection in the original variable space due to the penalization of the log-likelihood and feature selection in the transformed variable space when choosing the number of components in the regression model. Details about and comparisons of the various regularization methods may be found in [28], [29] and in Methods.

**Table 1:** Characteristics of the five regression regularization methods.

Method	Dimension reduction	Penalization	Variable selection
PCR	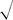	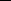	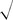 (on transformed variables)
PLS	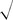	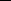	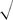 (on transformed variables)
SPLS	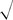	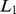	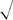
LASSO	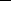	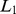	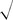
RR	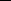	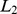	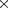

Since the extreme rarity of most resequencing variants could lead to computational and inferential challenges with feature selection and extraction methods, we also implemented and investigated adaptations of these methods specifically for rare variant analysis. Each method is implemented using two different model choice criteria, both with and without our rare variant adaptation. Using genetic variants identified by Sanger sequencing on three genes in 1998 individuals, we simulated phenotypes under a range of models, and then compared the ability to identify the causal variants using these regression regularization methods. We have also compared performance with three popular methods recently developed for rare variant analysis: the weighted count of Madsen and Browning (WE) [8], the variable threshold method (VT) [30], and the sequence kernel association test (SKAT) [9]. Many methods have been developed for rare variant analysis; we chose these methods for comparison since they represent both the burden methods and the variance-based methods, and have been shown to have good power [7].

In fact, we show that RR, PLS, LASSO and sparse PLS usually outperform WE, VT and SKAT as long as the causal variants are not singletons or extremely rare. Our comparison is timely, since there is great interest in methods for rare variant detection. One additional advantage of feature selection methods is that they can not only identify associations, but can also point towards which variants are likely the truly-associated ones.

## Results

Commonly-used methods for rare variants often pool rare alleles and fit simple regression models relating the phenotype to rare allele counts. However, the choice of threshold below which a variant is pooled or collapsed for rare-variant analysis is, of course, arbitrary. Although a 1% threshold is the traditional standard for differentiating between a polymorphism and a mutation [31], this may not be the optimal threshold for rare variant analysis.

In contrast, we are using multiple regression methods to look for rare variant associations. The five statistical models for feature selection and extraction were fit using well-known R packages [32] (see Methods). However, in each of these models, consideration must be given to model size. For the penalty methods, this is achieved by choosing the penalty parameter 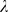. For the feature extraction methods, we chose the number of features to enter the model by using three well-known approaches for model selection or choice, AIC [33], BIC [34] and GIC [35]. (See Methods for details.)

To combine our chosen multiple regression methods with the concepts of pooling and collapsing, we propose an approach motivated by the variable threshold idea [30]. We defined a set of thresholds for defining rarity, starting at 5% and including all minor allele frequency observed (MAF) values smaller than this. For each threshold, we created a new variable that contained the unweighted count of minor alleles for all variants with MAF below the threshold, and we then added the entire set of new variables to the set of variables being analyzed. Hence, we have combined the feature selection methods with a generalized pooling strategy, and we have evaluated the performance of these hybrid approaches for detection of rare genetic variants.

For our evaluations, we used genotype data on three genes where the exons and flanking regions were Sanger sequenced in 1,998 individuals (courtesy of GlaxoSmithKline (GSK)) [36], [37]. We then simulated phenotypes following six simulation scenarios based on these genotypes. For simplicity, the missing values were imputed independently at each variant by randomly generating the missing genotype using the computed MAF. The three genes sequenced (anonymized data, called genes A, B and C) had respectively 98, 28 and 122 variant sites.

Continuous phenotypes were generated assuming a normal distribution 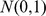 among individuals not carrying any causal genetic variants. From each gene, some rare variants (and possibly some common variants) were selected to be associated with the phenotype, and for carriers of these variants, the normal distributions were shifted. In our first set of simulations (Scenario set I), the shift is independent of allele frequency; however in a second set of simulations (Scenario set II), the size of the effect of the causal variants depends inversely on the MAF. The parameters used in the simulations are described in Table 2, and more details about the simulation design are given in Methods.

**Table 2:** Parameters for Simulation Scenarios.

Scenario	Causal rare variantthreshold	Direction of effect	Percentage of variants that are causal	Average effect size in standard deviations
**Scenario set I: Effect size constant across MAF**				
**I.1** Large10	MAF 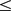 0.01	deleterious	10% rare	1.64
**I.2** Rare/Common	MAF 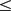 0.01	deleterious	4 rare, 4 common	1.64, 0.07
**I.3** Modest10	MAF 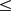 0.01	deleterious	10% rare	1
**I.4** Modest20	MAF 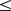 0.01	deleterious	20% rare	1
**I.5** Birectional	MAF 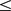 0.01	7.5% deleterious, 7.5% protective	15%	1.64, 1.64
**I.6** VeryRare	MAF 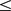 0.001	deleterious	20% rare	1.64
**Scenario set II: Effect size dependent (inversely) on MAF**				
**II.1** Large10	MAF 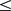 0.01	deleterious	10% rare	1.64
**II.2** Rare/Common	MAF 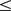 0.01	deleterious	4 rare, 4 common	1.64, 0.07
**II.3** Modest10	MAF 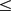 0.01	deleterious	10% rare	1
**II.4** Modest20	MAF 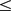 0.01	deleterious	20% rare	1
**II.5** Birectional	MAF 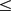 0.01	7.5% deleterious, 7.5% protective	15%	1.64, 1.64
**II.6** VeryRare	MAF 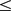 0.001	deleterious	20% rare	1.64

When fitting the models, a single measure of model fit was chosen for each method, after choosing all the parameters of the model (see Methods). Empirical power was calculated by comparing this test statistic to its distribution under 1000 permutations. In the analysis of permuted data, the parameters controlling model size and complexity were chosen independently within each permutation.

### Labelling and Nomenclature

**Figure 2:**
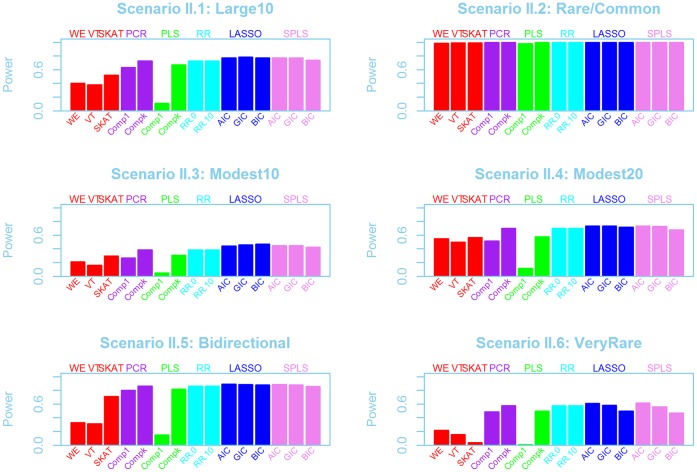
Test power for gene A with 98 variant sites under simulation scenarios I.1 to I.6. Power is shown for several different methods, including several options within each of the regularization methods. WE, VT and SKAT are shown in red, PCR in purple, PLS in green, RR in turquoise, LASSO in royal blue and SPLS in pink. Simulation scenarios are shown in Table 2.

**Figure 3:**
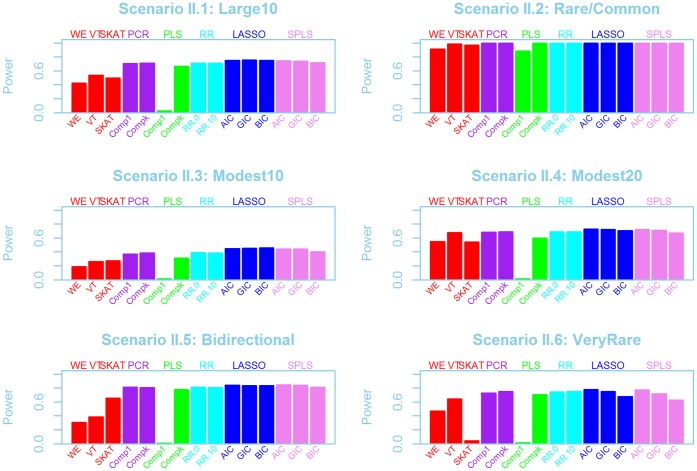
Test power for gene C with 122 variant sites under simulation scenarios II.1 to II.6. Power is shown for several different methods, including several options within each of the regularization methods. WE, VT and SKAT are shown in red, PCR in purple, PLS in green, RR in turquoise, LASSO in royal blue and SPLS in pink. Simulation scenarios are shown in Table 2.

Each of our five chosen methods was used to analyze 1000 simulated data sets, and the results are used to calculate empirical power at significance level 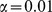. Permutation was used to assess significance for all methods, since the feature selection inherent in each method will lead to biased estimates of significance using asymptotic techniques. Using QQ-plots, the distribution of the empirical p-values under the null hypothesis is demonstrated in Figure S6 for most of the methods. Variability is within the expected error bounds. Power comparisons across the different methods are illustrated in Figure 2 for scenario set I and gene A, and in Figure 3 for scenario set II and gene C. Additional results (for genes B and C from Scenario set I, and for genes A and B from Scenario set II) are in Figures S1, S2, S3, and S4. Results in numeric form are also given in Table S1 and Table S2.

Methods are colour coded and labelled across the top of each figure. Several different options were used for fitting each of the multiple regression methods. For PCR and PLS, models with one component are denoted “Comp1”. The label “Compk” denotes models with 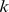 components, where 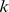 represents the number of features that explained 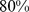 of the variance in the response. For RR, 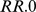 represents ridge regression with 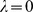, or equivalently an ordinarily linear regression. 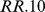 implies RR with a penalty parameter of 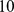. For the LASSO and SPLS methods, the labels AIC, BIC, or GIC indicate the method used for selecting penalty parameters. Finally, if the label terminates with “.p”, as in “Comp1.p” or “AIC.p”, then the pooled rare variant set was added to the set of predictor variables.

### Power Comparisons

With a few exceptions, any of the 5 multiple regression methods had better power than the three approaches developed specifically for rare variant analysis (WE, VT, SKAT), and furthermore, performance was often very similar across different variants of the multiple regression methods.

Consider first two situations where causal variants had clear large effects (I.1:Large10 and I.5:Bidirectional in Figure 2 for gene A, and Figure S2 for gene C). In these scenarios, the best powers of each of the 5 regularization methods were very comparable. A few specific choices for feature selection performed poorly: notably PCR with only one component showed poor power, as did PLS with one component (particularly when the pooled rare variants were included in the predictor space). LASSO, RR and SPLS showed very similar powers, and neither the variable selection technique nor the addition of pooled predictor variables altered power in these cases. When the effect size depends on MAF (Figure 3 and Figure S3), there is comparable performance for all multiple regression methods (apart from PCR and PLS with one component), and more power than VT, WE or SKAT.

When the causal variants had smaller effects (I.3:Modest10, I.4:Modest20 in Figure 2, similar models in Figure 3), there is slightly more variability between the different multiple regression methods. The LASSO, in particular, seems to have better power than other approaches. When 20% of the rare variants were causal, WE or VT sometimes had good power too.

With a mixture of rare and common variants, the LASSO again had better power than most other multiple regresion methods. Comparing this scenario across the three genes for scenario set I (Figures 2, S1 and S2), WE, VT and PCR had the best power for gene A but not for genes B and C. It seems that the common causal variants are aligned with the rare causals such that the first principal component captures the association identified by the burden methods.

When the effect size depends on the MAF (Scenario set II), the relative performances of the multiple regression methods were similar to Scenario set I; we saw very little alteration in the relative performances of the various algorithms. The multiple regression methods continued to perform well in comparison with VT, WE and SKAT. However, all methods had excellent power for the models with a mixture of rare and common variants (I.2 and II.2). This is due to the definition of the effect sizes in this scenario, where the average effect size was defined across all causal variants (see Table 2 and Methods).

In scenario I.6 and II.6, only variants with frequency less than 1/1000 were selected as causal. Power is substantially lower in this situation for all methods and the patterns of performance differ. In Scenario set I (Figures 2, S1 and S2), PCR (with one component and pooled predictors), PLS or RR have better power than LASSO and SPLS. In addition, VT performs well in this context for genes B and C. In contrast, when the magnitude of the effect is inversely dependent on MAF (scenario set II), all the multiple regression methods perform comparably to or better than VT; in particular, the LASSO or SPLS with AIC show good performance. It is interesting to note that SKAT performed very poorly in this scenario.

The addition of pooled variables to the predictor space did not seem to alter power in the simulations using Scenario set I. However as previously noted, PCR with one component and pooled predictor variables performed better than other approaches in scenario I.6:VeryRare, and is presumable capturing the causal rare variants through one or more of the pooled variables. Since in most cases, the pooled predictors made no difference, the figures for Scenario set II do not include pooled predictors.

When effects could act in both directions (I.4) VT and WE did very poorly, but these approaches are known to look only for variants acting in one direction [7], [11]. In contrast, the power of SKAT is much better than WE and VT in the bidirectional situation, since this test looks for changes in variances rather than means. Nevertheless, the regression-based approaches all have greater power than SKAT. As the percentage of variants that are causal increases, it has been shown that SKAT loses power relative to VT and WE [7], but this parameter has a less important effect on power than the effect size or the causal MAF distribution.

Gene B contains only 28 variant sites, and as a result the power for detecting association is low for all methods (Supplemental Figures S2 and S3). Our multiple regression implementions perform just as well (or just as poorly) as WE, VT or SKAT for this gene. Power is slighly better when the model includes a mixture of both rare and common variants (I.2 and II.2), and then the multiple regression methods perform better than the rare variant methods.

Finally, we did not see consistent changes in performance when comparing variable selection using AIC, BIC or GIC for the LASSO and SPLS. There are a couple of situations where power seemed better when using AIC, and other situation were BIC or GIC appeared to be the best. Given that these power estimates are based on 1000 simulations, the standard error of the power estimates is 1.6% or less, depending on the magnitude of the power.

### Causal Variable Identification

**Figure 4:**
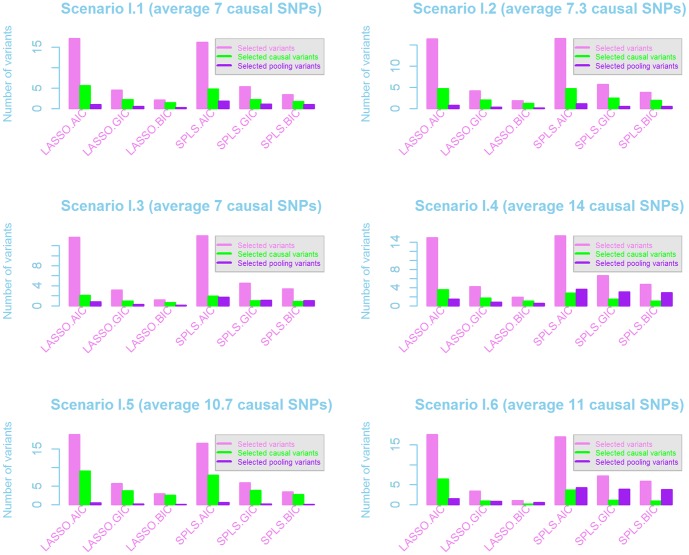
Number of variants selected by the LASSO and SPLS methods for Scenario set I. Numbers of variants selected are averaged across the three genes and across the simulations for AIC, GIC and BIC variable selection techniques. Within each set of three bars, the left (pink) bar shows the total number of variants selected, the middle (green) bar shows the number of causal variants selected, and the right bar (purple) shows the number of pooled variables selected.

After identifying whether a gene is associated with a phenotype, there is interest in finding which variants are strongly associated. Pooling and collapsing rare variant methods do not provide this kind of information. However, parameter estimates from the LASSO and SPLS methods can be helpful for this inference, since the final models can be examined to see whether the true causal variants were selected and retained. Figure 4 demonstrates whether the truly-associated variants were captured by LASSO or SPLS in the simulations using Scenario set I, averaged over the three genes. This figure shows three different aspects of variable selection. Firstly, the left (pink) bar in each set shows the the average numbers of variants selected across the 1000 simulations. In the centre (green) bar of each set is the average number of causal variants selected by the LASSO and SPLS methods. Finally the third (purple) bar shows the number of the pooled variables included in the final models.

AIC variable selection included more variables than BIC or GIC, for both LASSO and SPLS, and included a large proportion of the causal variants in each generating model. However, these AIC methods also selected a large number of non-associated variants. The number of non-associated variants was much smaller for BIC or GIC, but as a consequence, many truly-associated variants were missed. Power does depend on the degree of penalization, and when there is insufficient penalization or too many variants in the regression models, the power tends to be lower (Figure S5). Usually only one or fewer pooled variables were retained in the models, but occasionally there could be 2 different pooled variables (with different thresholds) kept in the same model (Figure 4).

### Adiponectin and ADIPOQ

Adiponectin levels are controlled by the ADIPOQ gene, and genetic variants in this gene are known to influence adiponectin levels [38]. Therefore, we compared the results for our tests of association between rare genetic variants at ADIPOQ and adiponectin in two data sets. The first data set included individuals from Twins UK data [39], [40] who were selected from the extremes of a pain phenotype; 175 out of 500 samples have available adiponectin values and had undergone exome sequencing. The exomes were captured by Nimblegen technologies and were resequenced by Beijing Genomics Institute (BGI) (unpublished data); 5 rare variants were identified in the gene and were analyzed together. The second analysis included 1375 individuals, again from the Twins UK data who were explicitly genotyped at two rare variants within ADIPOQ, and the analysis included only these 2 variants (unpublished data). These two SNPs were genotyped by Taqman in KBioscience, UK; 113 individuals were in both analyses.

Table 3 shows the results of the analyses of these two data sets. For the small data set of 175 individuals, several methods give rise to estimates of significance near 0.05, but among these, PCR and RR with a penalty of 10 show the smallest p-values. The WE method shows no relationship with phenotype in this context. Three of the 5 SNPs showed some univariate association with adiponectin. When using SPLS with AIC, all three of these markers were included in the chosen model. For LASSO with AIC, two of the three markers were selected. When using GIC or BIC, the marker with the strongest univariate significance was always included. In the analysis of the larger data set, all of the methods showed strong significance with the minimum possible p-value for 10^6^ permutations, and the p-values obtained are smaller than the p-values for the comparison methods WE, VT or SKAT. Both variants were included by the LASSO and SPLS methods.

## Discussion

Resequencing efforts identify many extremely rare or private genetic variants, often of unknown function. Analysis of association of such variants is difficult due to the sparsity of the data. Although any statistical inference about an event seen only once is impossible, we hypothesized that modern multiple regression methods might be able to find some associations between high-dimensional sparse data and phenotypes, and we demonstrated that this is, in fact, the case using Sanger sequencing data on almost 2000 individuals at three genes. Our analysis of adiponectin and the ADIPOQ locus confirmed this potential for increased power using multiple regression methods. Furthermore, we showed that we have the ability to identify a substantial proportion of the causal variants within each gene.

Performance of the multiple regression methods was not as good in a few situations. Dimension reduction methods (PCR, PLS) with only one component tended to perform poorly. In fact, this could probably be expected. These methods are normally used together with a rule for determining the best number of components, and so choosing only one component is not the normal implementation. Multiple regression methods also performed poorly in a simulation where the causal variants were very rare; in that situation there was a large discrepancy between the frequency of the causal variants (0.001) and the largest threshold we used for pooling rare alleles (0.05). Therefore, refining our combined regression and pooling approach may improve performance in this case. For example, we could reduce the number of MAF thresholds that we included, or use counts weighted by the inverse of MAF.

It is difficult to understand why some models would include multiple pooled variables with different thresholds, as was seen in Figure 4. Due to the rarity of the variants, many of the pooled variables may be highly collinear with the original data as well as with other pooled variables. We believe this is why the performance of the models with the pooled predictors was so similar to the performance without this set of variables. Furthermore, the selection of more than one pooled variable may also suggest that pooled variables at distinct allele frequency intervals could be useful.

Since permutations are required, the computational overhead of some of these regression approaches can be quite high. Supplemental Table S3 shows average run times on a 2.8 GHz blade, and demonstrates that PLS, LASSO and SPLS are much slower than the other methods. Taking the largest of our 3 genes, the LASSO would need 108 days of computer time to do an exome-wide analysis of 20,000 genes, with 1000 permutations. This would require the use of a processor with multiple compute nodes. In contrast, for RR, only 27 hours of CPU time would be needed to use this method with our code. Computation times could be substantially improved by using an incremental number of permutations, with more precision for smaller p-values.

It is interesting to speculate on why these methods work. Some of the rare causal variants in our simulations could have been observed in up to 20 individuals out of the 2000 in the sample, and hence there would be adequate power to identify these variants. It is also possible that the presence of long-range correlation between variants could provide information to PCR or PLS methods. Long-range correlation between common and rare variants can occur due to patterns of ancestral recombinations and co-occurrence of rare variants in some parts of a gene genealogy [41]. Power was lower across all methods for the smallest gene, but this gene contained only 2 variants with MAF over 1%, and so our strategy may have been unable to capture additional signals through linkage disequilibrium.

Nevertheless, these multiple regression methods were not designed for data as sparse as data from resequencing studies. We therefore proposed augmenting the variable space with new variables defined by pooling rare variants together. Instead of choosing a single threshold, we augmented the predictor space by a set of pooled counts based on a range of thresholds. These variables would, of course, be highly correlated with each other. We did not find an improvement in power in most situations when we added the pooled variables, and we believe that this is due to the increase in the parameter space, so that the models had more difficulty identifying the best predictors.

**Table 3:** P-values for association tests between Adiponectin levels and the ADIPOQ gene.

Method	175 samples, 5 SNPs	1376 samples, 2 SNPs
WE	0.7419	0.0130
VT	0.0736	0.0006
SKAT	0.1596	8×10^−6^
PCR	0.0158	10^−6^
PLS	0.0290	10^−6^
RR *λ* = 0	0.0290	10^−6^
RR *λ* = 10	0.0780	10^−6^
LASSO	0.0846	10^−6^
SPLS	0.0892	10^−6^

Note: 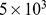 and 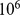 permutations were used for the two datasets, respectively, to obtain empirical significance levels. PCR and PLS were fitted using only one component. When multiple components were used, the p-values were very similar. AIC was used to select model size for LASSO and SPLS.

Any division of genetic variants into “common” and “rare” is arbitrary. Our choice of 5% for the upper threshold of rarity for pooling may also have an impact on the performance of the models including pooled variable sets. It would be interesting to investigate whether considerations of population history could be used to set more appropriate thresholds distinguishing rare variants from common ones.

Simulations can be designed to favour one analytic strategy over another. When we modelled a constant effect of the causal variants (Scenario set I), as expected the VT method usually outperformed the WE method. In contrast, in Scenario set II where the effect depended on MAF, performance of WE was improved. Our simulations did not explicitly select correlated genetic variants to be causal, and therefore the dimension reduction approaches of PCR and PLS would not necessarily be expected to outperform other approaches. However, the idea that a small percentage of the rare genetic variants in a gene are causal underlies most of the methods developed for rare variant analysis, and therefore the better performance of multiple regression methods is a pleasant surprise.

All simulations were based on the genotype data from three Sanger sequenced genes. The variants chosen as causal were randomly selected for each simulated data set and therefore there was variability across the simulations in the rarity of the causal variants. However, evaluation of performance on a larger set of genotypes may provide additional insight into performance of these methods.

Predictions of changes in amino acids in proteins, or predictions of sequence conservation have been used with success to distinguish potentially causal variation from variation that is unlikely to be causal [30]. Any of the methods evaluated here could be combined with such predictions to improve the variable selections, by implementing an appropriate weighting of the variants. Similarly, covariates could easily be included into these multiple regression models. However, our current implementation measures the strength of the genetic association through a global model fit statistic; this would therefore need alteration so that the summary statistic excludes the effects of covariates and focuses only on the genetic variants.

Theoretically, such methods could simultaneously model all the genetic variants in more than one gene, such as all variants in a pathway, or conceivably all exome-identified variants. Genome-wide simultaneous modeling has been suggested by several authors, in particular using Bayesian methods [42]. We have not attempted such models using these multiple regression methods, however, we anticipate that the penalty needed as a result of the substantial model selection steps would overwhelm power.

We obtained excellent power over a variety of simulation scenarios with many of our implementations of these multiple regression methods. Therefore, the choice of best method may be partially the preference of the data analyst. PLS or SPLS methods may be beneficial for modelling jointly covariates and genotypes. We like ridge regression since it is computationally fast, but where computation time is less of a concern or the variable selection aspect is of importance, the LASSO could be an excellent choice since the power is often slightly better.

### Conclusions

Methods developed for high-dimensional data may outperform other approaches for rare variant analysis. These methods will simultaneously model the effects of all genetic variants in a gene, common or rare. Furthermore, unlike collapsing, counting, or variance-based methods for rare variant association analysis, some of these regression methods can identify the most likely causal variants.

## Methods

### Ethical Statement

The genotype data used for our simulations represents a re-use of data and no new human interventions were conducted. No additional IRB approvals were sought for the simulation studies. The Committee on Ethics in Clinical Research, CHUV, Lausanne University, Lausanne, Switzerland approved the original protocols for sample collection for the genotype data used in simulations. All participants in Twins UK provided informed written consent, and the research protocol was approved by institutional ethics review committees at Kings College London. Again, the data used for our analyses represents a re-use of data that has been previously analyzed and no further IRB approvals were sought.

### Model Details

Suppose we have a sample of 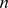 independent individuals who have been sequenced to identify genetic variation in at least one candidate gene, and measured for a continuous trait. Assume that equation (1) describes the true relationship between the phenotype and genotypes, where the set of genotypes includes all identified locations that vary between individuals in the sample. We fit several multiple regression methods including variable selection or feature extraction methods. These methods have been previously compared, but not for analysis of rare genetic variants [43]. An R package (RVtests) containing the implementation of the tests described below is available from the authors or at www.mcgill.ca/statisticalgenetics/.

### Feature Extraction Methods

In PCR [24], the original predictor variables 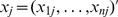, 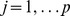, are transformed to principal components that explain variance in the predictor space, without considering the relationship to the response variable. Specifically, let 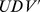 be the singular value decomposition of 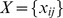. Then the fitted response for PCR with 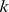 components is 
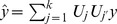
 [29], where 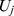 is the 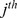 column of 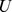, and 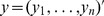.

In PLS [25], [26], orthogonal scores are created that have both high variance and high correlation with the response, 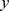. Let 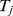, 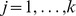, be the orthogonal scores in a PLS model with 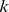 components. Then the fitted response can be written 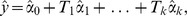 where 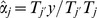.

The fitted values for PCR and PLS depend on the number of components, 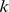, which is also referred to as the size of the model. Although 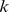 is often chosen by the use of cross-validation, in the rare variant context, we wanted to identify algorithms that are computationally efficient, and therefore, we compared performance for two values of 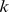, 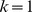, and 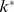, where 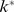 is chosen so that 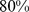 of variance in the response is explained by the model. We also fit models with a large value of 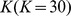, but results were not as good and are not shown. All our simulations were implemented with the R statistical programming language [32]. We used the R package ‘pls’ [44] for getting the PLS scores, 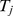, and R function ‘svd’ for calculating the PCR scores, 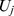.

### 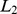 Penalization Method (Ridge Regression)

Parameters in RR models [22] are shrunk towards zero by adding to the regression model a penalty parameter which is a function of the squared regression coefficients, i.e., 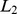 norm. Following the notation used above, where 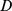 is a matrix containing the singular values of 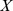, and 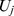 are the singular vectors, the RR fitted response is 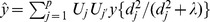 [29], where 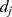 is the 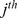 diagonal element of 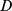. The penalty parameter 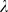, where 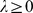, controls the degree of shrinkage; for large values of 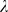 all parameters become close to zero and the effective dimension of the model is reduced. In contrast, when 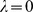, RR reduces to an ordinary linear regression model. Results are shown for 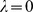 and 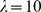. We also completed simulations with 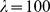 but performance was comparable to 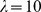 and results are not shown. We used the correlation between the observed 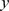 and the fitted values 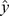, 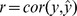, as our measure of goodness of fit.

### Methods Using 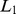 Penalization

The penalty parameter in LASSO [23] and SPLS [27] can be chosen by classic model selection criteria [45], [46]. Here we used AIC [33], BIC [34], and GIC [35] to choose this parameter. For SPLS, a series of models was fit, varying the number of hidden components 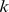 between 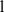 and 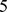, as well as the thresholding parameter 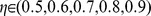. The best model choice over the two-way grid of parameter values was chosen by AIC, BIC or GIC.

To evaluate model performance for LASSO and SPLS, we used the selected final model F-test p-value as the score measuring model performance. The R-package ‘glmnet’ was used for LASSO [47] and the package ‘spls’ for SPLS [27].

### Pooling Rare Variants

Let 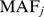, 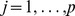, be the MAF of the 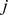-th variant. For a chosen threshold 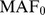, the set of rare variants can be defined as the variants 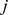 with 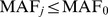, and a pooled rare variant count is 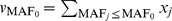, where 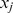 is the number of minor alleles at variant 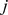. To combine the regression methods with the concepts of pooling and collapsing, we propose an approach motivated by the variable threshold idea [30], and so we defined a set of thresholds for defining rarity, starting at 5% and including all MAF values smaller than this. Let 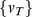 be a new set of variables 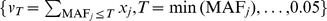 that pool rare variants for a series of possible thresholds, 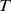, on the minor allele frequency. These new sets of variables were added as possible predictor variables in the regression models. Since we did not identify any benefit to including these pooled variables in the set of predictor variables in Scenario set I, the simulaton results for Scenario set II are presented without the inclusion of these additional predictors.

### Study Sample

The subjects used in this paper are a subset of the CoLaus study, a population-based study of 6,188 Lausanne residents aged 35 to 75 years [37].

### Sanger Sequencing Data

Sanger sequence data for the exons and flanking regions of three genes from 1,998 individuals were provided by GlaxoSmithKline (GSK) [36]. Missing values of each rare variant were imputed independently from others based on the computed MAF, as in [7]. All non-polymorphic base-pair markers were removed from the sequence data. The three genes used in our simulations contained, after removal of monomorphic variants, 98, 28 and 122 variant sites, respectively. Of these, 85, 26 and 99 variants, respectively, were seen at allele frequencies less than 1%. Coding lengths for these genes were 4094, 1239 and 1500 base pairs.

### Phenotype Simulation

Within each simulation, a proportion of the rare variants was randomly selected to be causal, depending on the simulation scenario (Table 2). The threshold for “rare” is given in the second column of Table 2; all variants with MAF below the threshold could be chosen to be causal in any simulation. The phenotypes were generated from a 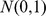 distribution for individuals not carrying any rare variants. In Scenario set I, for carriers of one or more rare variants, the phenotype was assumed to be distributed as 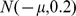 where the values of 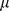 are given in the last column of Table 2. For Scenario set II, we define the effect of each variant as 
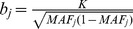
 for a chosen constant 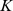. This constant was chosen so that the average effect of all 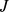 causal variants, 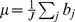, has the values shown in Table 2. For individuals carrying more than one causal rare variant, the phenotype was drawn from the normal distribution with mean corresponding to the most rare causal variant carried by that individual. For scenarios I.5 or II.5, where the effects could be bidirectional, the mean 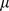 could be either positive or negative. Similar simulation parameters were used by [7].

### Permutations

For each simulated data set, the phenotype data was permuted relative to all the genotype data and the analysis was repeated. For each permutation, the analysis included all model fitting steps, so that variable selection or identification of the best model as a function of AIC or BIC was repeated for each permutation step. Using the chosen measure of model fit for each method (described above), we then compared this statistic between the permuted data sets and the original simulated data set, and counted the number of permutations where the model fit statistic was more extreme than in the original data.

### Software

An R package, RVtests, that uses these approaches to test for rare variant associations, is available from the authors or from cran-r.project.org.

## Supporting Information

Figure S1 **Power for Scenario set I for gene B.**
(PDF)

Figure S2 **Power for Scenario set I for gene C.**
(PDF)

Figure S3 **Power for Scenario set II for gene A.**
(PDF)

Figure S4 **Power for Scenario set II for gene B.**
(PDF)

Figure S5 **Power for the LASSO method as a function of the penalty parameter.**
(PDF)

Figure S6 **QQ plot of empirical p-values for gene C under the null hypothesis.**
(PDF)

Table S1 **Test power of WE, VT, SKAT, PCR, PLS, RR, LASSO, and SPLS for three genes and for scenario set I where variant effects do not vary with the minor allele frequency.**
(PDF)

Table S2 **Test power of WE, VT, SKAT, PCR, PLS, RR, LASSO, and SPLS for three genes and for scenario set II where variant effects vary with the minor allele frequency.**
(PDF)

Table S3 **Simulation run time for three genes and six scenarios.**
(PDF)
