# Supplementary material for: Multiple Regression Methods Show Great Potential for Rare Variant Association Tests
Source: PLoS One. 2012 Aug 8;7(8):e41694. doi: 10.1371/journal.pone.0041694 (PMC3420665; doi:10.1371/journal.pone.0041694)
Supplement: Figure S3 — Power for Scenario set II for gene A. (PDF) [file pone.0041694.s003.pdf]

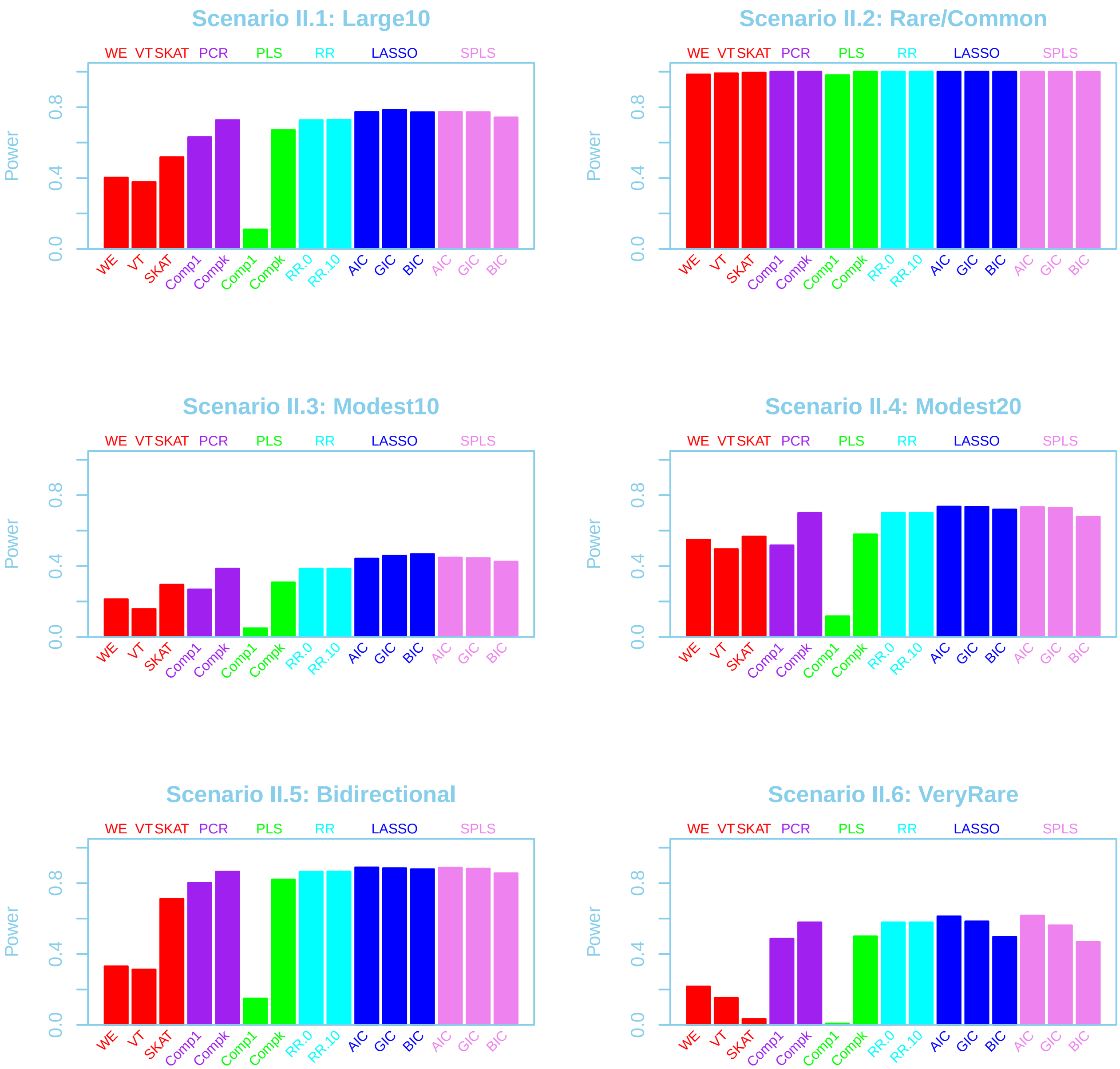

Figure S3. Power for Scenario set II for gene A. Power is shown for several different methods, including several options within each of the regularization methods. WE, VT and SKAT are shown in red, PCR in purple, PLS in green, RR in turquoise, LASSO in royal blue and SPLS in pink. Scenarios II.1 to II.6 are described in Table 2. Suffix "k" imples that the number of components k was chosen to explain 80% of the variance.
