# Supplementary material for: Multiple Regression Methods Show Great Potential for Rare Variant Association Tests
Source: PLoS One. 2012 Aug 8;7(8):e41694. doi: 10.1371/journal.pone.0041694 (PMC3420665; doi:10.1371/journal.pone.0041694)
Supplement: Figure S5 — Power for the LASSO method as a function of the penalty parameter. (PDF) [file pone.0041694.s005.pdf]

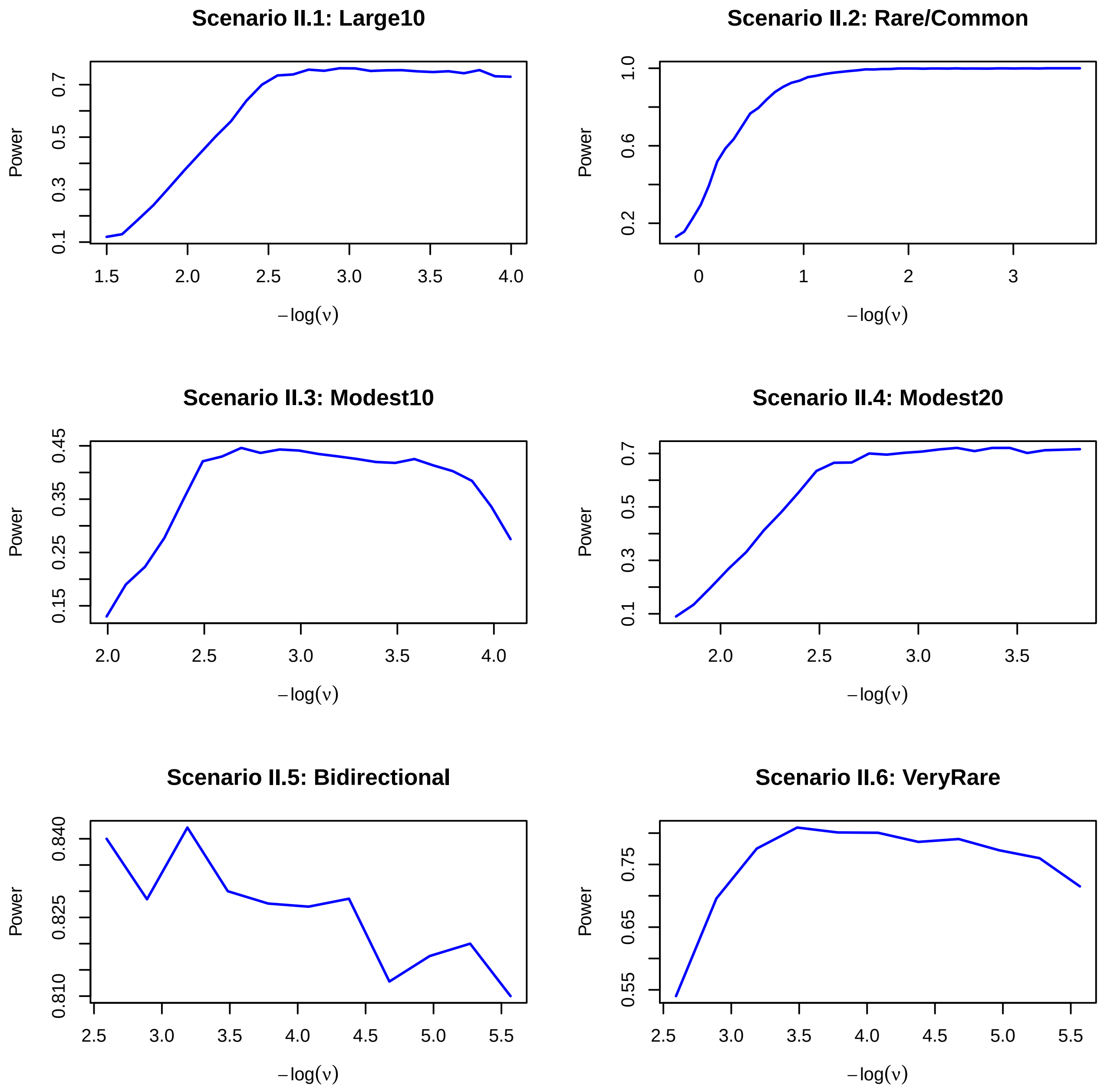

Figure S5. Power for the LASSO method as a function of the penalty parameter (labelled here as ν) for gene C and Scenario set II.
