# Supplementary figures and images for: Multiple Regression Methods Show Great Potential for Rare Variant Association Tests
Source: PLoS One. 2012 Aug 8;7(8):e41694. doi: 10.1371/journal.pone.0041694 (PMC3420665; doi:10.1371/journal.pone.0041694)

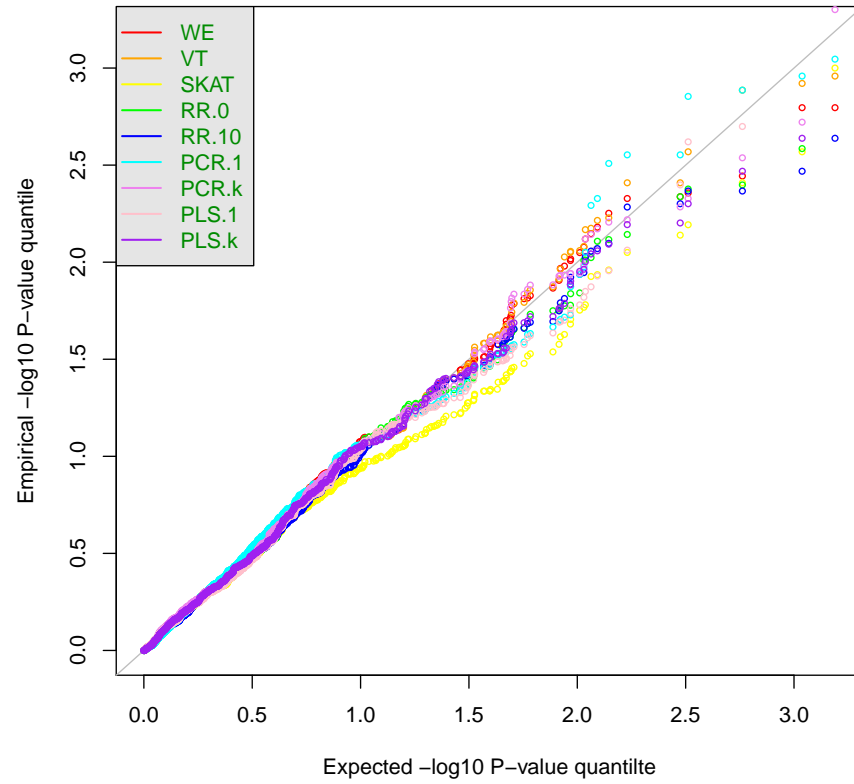

Figure S6. QQ plot of empirical p-values for gene C under the null hypothesis.

Supplement: Figure S6 — QQ plot of empirical p-values for gene C under the null hypothesis. (PDF) [file pone.0041694.s006.pdf]
