# Supplementary material for: Multiple Regression Methods Show Great Potential for Rare Variant Association Tests
Source: PLoS One. 2012 Aug 8;7(8):e41694. doi: 10.1371/journal.pone.0041694 (PMC3420665; doi:10.1371/journal.pone.0041694)
Supplement: Table S1 — Test power of WE, VT, SKAT, PCR, PLS, RR, LASSO, and SPLS for three genes and for scenario set I where variant effects do not vary with the minor allele frequency. (PDF) [file pone.0041694.s007.pdf]

Table S1: Test power of WE, VT, SKAT, PCR, PLS, RR, LASSO, and SPLS for three genes and for scenario set I where variant effects do not vary with the minor allele frequency.

				PCR				PLS				RR				LASSO						SPLS					
Scenario	WE	VT	SKAT	1	pool	k^*	pool	1	pool	k^*	pool	1	pool	10	pool	AIC	pool	GIC	pool	BIC	pool	AIC	pool	GIC	pool	BIC	pool
(Gene A)																											
1	0.40	0.27	0.59	0.08	0.39	0.66	0.72	0.56	0.04	0.74	0.72	0.74	0.70	0.74	0.72	**0.78**	0.77	0.77	0.76	**0.78**	0.76	0.77	0.77	0.75	0.75	0.73	0.74
2	0.87	0.91	0.56	**0.90**	0.29	0.67	0.70	0.06	0.07	0.64	0.65	0.64	0.63	0.64	0.65	0.76	0.74	0.75	0.74	0.79	0.78	0.70	0.69	0.67	0.66	0.66	0.65
3	0.17	0.06	0.23	0.04	0.12	0.11	0.15	0.09	0.02	0.16	0.15	0.16	0.14	0.16	0.16	0.27	0.26	0.30	0.30	0.35	**0.36**	0.27	0.26	0.30	0.29	0.29	0.30
4	0.62	0.37	0.55	0.09	0.62	0.28	0.52	0.23	0.00	0.50	0.51	0.50	0.43	0.50	0.51	0.59	0.59	0.62	0.63	0.71	0.70	0.60	0.60	0.63	0.63	0.61	0.61
5	0.09	0.09	0.79	0.08	0.04	0.92	0.93	0.85	0.61	**0.94**	**0.94**	**0.94**	0.93	**0.94**	**0.94**	0.93	**0.94**	0.91	0.92	0.90	0.92	**0.94**	**0.94**	0.92	0.92	0.91	0.91
6	0.24	0.06	0.02	0.00	0.45	0.29	0.38	0.31	0.01	0.36	0.36	0.36	0.31	0.36	0.36	0.25	0.24	0.09	0.09	0.04	0.04	0.23	0.23	0.08	0.08	0.10	0.10
(Gene B)																											
1	0.18	0.25	0.19	0.03	0.10	0.24	0.28	0.28	0.21	0.28	0.28	0.28	0.27	0.28	0.28	0.28	0.30	0.27	0.29	0.25	0.28	**0.30**	**0.30**	0.29	0.29	0.27	0.27
2	0.49	0.51	0.27	0.21	0.22	0.46	0.50	0.53	0.35	0.53	0.55	0.53	0.53	0.53	**0.56**	0.53	0.52	0.50	0.48	0.46	0.43	0.52	0.52	0.48	0.48	0.43	0.43
3	0.07	0.14	0.12	0.01	0.03	0.06	0.14	0.12	0.10	0.12	0.12	0.12	0.12	0.12	0.13	0.11	**0.15**	0.11	**0.15**	0.11	0.14	**0.15**	**0.15**	**0.15**	**0.15**	0.14	0.14
4	0.19	0.30	0.21	0.01	0.10	0.10	0.24	0.23	0.17	0.24	0.23	0.24	0.22	0.24	0.23	0.21	0.24	0.21	0.24	0.21	0.24	0.24	0.24	0.24	0.24	0.23	0.23
5	0.02	0.10	0.26	0.05	0.01	0.41	0.41	**0.42**	0.38	**0.42**	0.43	**0.42**	0.40	**0.42**	**0.42**	0.38	0.40	0.35	0.36	0.33	0.34	0.39	0.39	0.37	0.37	0.34	0.34
6	0.14	**0.15**	0.01	0.02	**0.11**	0.08	0.07	0.09	0.05	0.10	0.08	0.10	0.07	0.10	0.08	0.07	0.04	0.04	0.01	0.01	0.01	0.04	0.04	0.02	0.02	0.02	0.02
(Gene C)																											
1	0.38	0.56	0.52	0.01	0.39	0.69	0.76	0.77	0.08	0.78	0.78	0.78	0.76	0.78	0.78	**0.80**	0.82	0.77	0.80	0.79	**0.80**	0.82	0.82	0.78	0.78	0.74	0.74
2	0.31	0.38	0.28	0.20	0.06	0.38	0.42	0.29	0.15	0.45	0.45	0.44	0.42	0.45	0.45	0.53	0.51	0.52	0.52	0.54	**0.55**	0.52	0.52	0.50	0.50	0.46	0.46
3	0.14	0.19	0.22	0.01	0.12	0.10	0.16	0.18	0.05	0.19	0.19	0.19	0.17	0.19	0.19	0.24	0.24	0.26	0.26	**0.31**	0.30	0.24	0.24	0.26	0.26	0.25	0.25
4	0.59	**0.74**	0.48	0.01	0.59	0.29	0.46	0.50	0.00	0.50	0.48	0.50	0.44	0.50	0.48	0.51	0.53	0.53	0.56	0.55	0.55	0.53	0.53	0.52	0.52	0.46	0.46
5	0.03	0.09	0.73	0.02	0.01	0.94	0.95	**0.96**	0.69	**0.96**	0.95	0.96	0.94	0.96	0.95	**0.96**	**0.96**	0.93	0.93	0.92	0.92	**0.96**	**0.96**	0.92	0.92	0.89	0.89
6	0.47	**0.66**	0.03	0.01	0.65	0.39	0.54	0.46	0.00	0.48	0.50	0.48	0.43	0.48	0.50	0.32	0.34	0.12	0.13	0.04	0.03	0.34	0.34	0.12	0.12	0.12	0.12
