# Supplementary material for: Multiple Regression Methods Show Great Potential for Rare Variant Association Tests
Source: PLoS One. 2012 Aug 8;7(8):e41694. doi: 10.1371/journal.pone.0041694 (PMC3420665; doi:10.1371/journal.pone.0041694)
Supplement: Table S2 — Test power of WE, VT, SKAT, PCR, PLS, RR, LASSO, and SPLS for three genes and for scenario set II where variant effects vary with the minor allele frequency. (PDF) [file pone.0041694.s008.pdf]

Table S2. Test power of WE, VT, SKAT, PCR, PLS, RR, LASSO, and SPLS for three genes and for scenario set II where variant effects vary with the minor allele frequency.

				PCR		PLS		RR		LASSO			SPLS		
Scenario	WE	VT	SKAT	1	k^*	1	k^*	1	10	AIC	GIC	BIC	AIC	GIC	BIC
(Gene A)															
1	0.40	0.38	0.52	0.63	0.73	0.11	0.67	0.73	0.73	0.77	**0.79**	0.77	0.77	0.77	0.74
2	0.98	0.99	0.99	1.00	1.00	0.98	1.00	1.00	1.00	1.00	1.00	1.00	1.00	1.00	1.00
3	0.21	0.16	0.29	0.27	0.38	0.05	0.31	0.38	0.38	0.44	0.46	**0.47**	0.45	0.44	0.42
4	0.55	0.50	0.57	0.52	0.70	0.12	0.58	0.70	0.70	**0.74**	0.73	0.72	0.73	0.73	0.68
5	0.33	0.31	0.71	0.80	0.86	0.15	0.82	0.86	0.87	0.89	0.88	0.88	**0.89**	0.88	0.86
6	0.22	0.15	0.03	0.49	0.58	0.01	0.50	0.58	0.58	**0.61**	0.58	0.50	0.62	0.56	0.47
(Gene B)															
1	0.20	0.26	0.20	0.33	0.33	0.06	0.31	0.33	0.33	0.39	0.40	**0.41**	0.39	0.39	0.38
2	1.00	1.00	0.99	1.00	1.00	1.00	1.00	1.00	1.00	1.00	1.00	1.00	1.00	1.00	1.00
3	0.08	0.15	0.12	0.17	0.17	0.03	0.14	0.17	0.17	**0.18**	0.18	0.18	0.18	0.18	0.17
4	0.20	0.29	0.20	0.28	0.28	0.04	0.21	0.28	0.28	**0.30**	0.30	0.30	0.30	0.30	0.28
5	0.13	0.17	0.28	0.46	0.46	0.06	0.42	0.46	0.46	**0.50**	0.50	0.46	0.50	0.50	0.46
6	0.14	0.16	0.01	0.24	0.24	0.02	0.22	0.24	0.24	**0.27**	0.25	0.23	0.27	0.25	0.22
(Gene C)															
1	0.42	0.54	0.50	0.70	0.71	0.02	0.66	0.71	0.71	0.75	**0.75**	0.75	0.75	0.74	0.72
2	0.91	0.98	0.97	1.00	1.00	0.88	1.00	1.00	1.00	1.00	1.00	1.00	1.00	1.00	1.00
3	0.19	0.26	0.27	0.37	0.39	0.02	0.31	0.39	0.39	0.45	0.45	**0.46**	0.44	0.44	0.40
4	0.55	0.68	0.54	0.68	0.69	0.01	0.60	0.69	0.69	**0.73**	0.72	0.71	0.72	0.71	0.67
5	0.31	0.38	0.66	0.81	0.81	0.01	0.78	0.81	0.81	0.84	0.83	**0.84**	0.84	0.84	0.81
6	0.47	0.65	0.04	0.73	0.75	0.01	0.71	0.75	0.75	**0.78**	0.75	0.68	0.77	0.72	0.62
