# Supplementary material for: Multiple Regression Methods Show Great Potential for Rare Variant Association Tests
Source: PLoS One. 2012 Aug 8;7(8):e41694. doi: 10.1371/journal.pone.0041694 (PMC3420665; doi:10.1371/journal.pone.0041694)
Supplement: Table S3 — Simulation run time for three genes and six scenarios. (PDF) [file pone.0041694.s009.pdf]

Table S3. Simulation run time for three genes and six scenarios (Unit: minutes per simulation).

	WE/VT	SKAT	RR	PCR	PLS	LASSO	SPLS
(Gene A)							
1	1.16	0.68	0.07	0.01	8.11	9.20	10.10
2	1.16	0.70	0.07	0.01	8.06	9.26	10.05
3	0.91	0.68	0.07	0.01	8.01	9.41	10.01
4	0.99	0.70	0.07	0.01	8.04	9.10	10.11
5	1.03	0.70	0.07	0.01	8.12	9.36	10.08
6	0.99	0.68	0.07	0.01	7.89	9.15	10.20
(Gene B)							
1	0.35	0.24	0.02	0.01	1.15	7.02	6.74
2	0.35	0.24	0.02	0.01	1.12	7.01	6.87
3	0.34	0.25	0.02	0.01	1.18	7.03	6.77
4	0.34	0.24	0.02	0.01	1.10	7.14	6.81
5	0.36	0.24	0.02	0.01	1.11	7.14	6.82
6	0.36	0.24	0.02	0.01	1.09	7.00	6.80
(Gene C)							
1	0.53	0.87	0.09	0.02	12.39	7.51	10.94
2	0.98	0.88	0.08	0.02	12.59	7.57	10.86
3	0.76	0.88	0.08	0.02	12.41	6.03	10.88
4	1.41	0.90	0.08	0.02	12.65	6.06	10.79
5	1.57	0.87	0.08	0.02	12.28	6.24	10.82
6	0.68	0.88	0.08	0.02	12.46	6.10	10.88

Note: WE/VT represents total run time for both WE and VT methods. All models were run on the Colosse cluster of Compute Canada which has 2.8GHz blades in groups of 8, with a total of 24Gb RAM per 8 blades. One set of 8 blades was used for analysis.
